# In-Lens Band-Pass Filter for Secondary Electrons in Ultrahigh Resolution SEM

**DOI:** 10.3390/ma12142307

**Published:** 2019-07-19

**Authors:** Ivo Konvalina, Filip Mika, Stanislav Krátký, Eliška Materna Mikmeková, Ilona Müllerová

**Affiliations:** Institute of Scientific Instruments of the CAS, v. v. i., Královopolská 147, 612 64 Brno, Czech Republic

**Keywords:** band-pass filter of signal electrons, SE detection, trajectory simulations

## Abstract

Scanning electron microscopes come equipped with different types of detectors for the collection of signal electrons emitted from samples. In-lens detection systems mostly consist of several auxiliary electrodes that help electrons to travel in a direction towards the detector. This paper aims to show that a through-the-lens detector in a commercial electron microscope Magellan 400 FEG can, under specific conditions, work as an energy band-pass filter of secondary electrons that are excited by the primary beam electrons. The band-pass filter properties verify extensive simulations of secondary and backscattered electrons in a precision 3D model of a microscope. A unique test sample demonstrates the effects of the band-pass filter on final image and contrast with chromium and silver stripes on a silicon substrate, manufactured by a combination of e-beam lithography, wet etching, and lift-off technique. The ray tracing of signal electrons in a detector model predicate that the through-the-lens detector works as a band-pass filter of the secondary electrons with an energy window of about 3 eV. By moving the energy window along the secondary electron energy spectrum curve of the analyzed material, we select the energy of the secondary electrons to be detected. Energy filtration brings a change in contrast in the image as well as displaying details that are not otherwise visible.

## 1. Introduction

Today’s scanning electron microscopes (SEMs) use an immersion objective lens (OL) to improve image resolution. A strong magnetic field penetrates to the sample region and decreases the aberration coefficients of the objective lens. The second effect of the magnetic field near the sample is a collimation of signal electrons to the optical axis. In this way, a large part of the signal electrons are guided into the OL, where they are processed further and detected. Thus, the final contrast of the SE images can vary with intensity and distribution of electrostatic and magnetic fields in the area where the signal electrons are moving [1]. 

The new detection systems, especially in-lens, offer a wide range of electron detection capabilities. The in-lens detector is placed inside the electron column of the microscope in association with auxiliary electrodes. Due to optimized parameters of the electrostatic and magnetic field near the sample and inside the column, the secondary electrons (SEs) are detected with high collection efficiency [2]. Changing the intensity of the electrostatic and magnetic fields in the space where the signal electrons are moving allows the detector’s preferences to be changed, especially concerning the energy of detected electrons and the polar angle under which electrons are emitted from the sample. Detectors at certain conditions operate as a filter of signal electrons. 

Low energy scanning electron microscopy (energy scale to the range from 5 keV down to, say, 50 eV) is one of the most popular methods for surface imaging and analysis. One important feature is the penetration depth of incident electrons, which shortens proportionally to energy. The lateral diffusion of electrons is also shorter and the interaction volume decreases [3]. Secondary electron imaging in the ultrahigh resolution SEM can be used to study the samples composition and its surface topography. 

Recently, great interest has been devoted to the filtering of secondary electrons to obtain additional information on the observed specimen [4].

The energy spectrum of emitted electrons depends on the material, the crystal orientation, and the surface layers. Suitably selected filtration of detected electrons can result in highlighting some sample properties, especially when we observe a combination of multiple materials. An energy-filtered image may contain more information than unfiltered. More details are visible for some samples. The contrast of the filtered image changes and new topography and material information can be observed [5]. 

The SE energy filtering in SEM has been widely used for many applications. In general, this method can be used as a technique for spectroscopy of low-energy secondary electrons emitted from many different materials [6,7] and for observing atomically thin films on metal substrates [8]. 

The energy filtering is very useful for the characterization of semiconductors, such as studying the image contrast of doped areas and semiconductor dopant mapping [9,10,11,12,13,14,15]. An energy selective scanning electron microscopy (ESSEM) can be used for the reduction of the effect of contamination layers on contrast [16]. 

The ultrahigh resolution energy filtered SEM will be able to help the understanding of complex, nano-structured organic materials [17].

For filtering secondary electrons in standard commercial electron microscopes, various add-on units are used. It is predominantly a system composed of a detector and filter grids, or a deflector, which is inserted into the microscope chamber. Add-on units are available in several variations. One option is a low-pass secondary electron detector [18] that can discriminate not only energy, but also the emission angle, of SEs. Another variant is the deflector/filter configuration [19] that acts as a band-pass energy filter whose energy pass range changes with deflector voltage. Compared to additional units, a through-the-lens detector in a field emission gun SEM, installed as standard in the objective lens, can be used for energy filtering of secondary electrons. Deflector voltage sets the low-pass filter [13]. 

Recently, the widely expanded in-lens detector allows for some filtration through the auxiliary electrodes that are in the column and help direct the electrons to the detector. 

In this paper, we focus on the microscope Magellan 400 FEG with Elstar column [20]. The Elstar column should be equipped by a new type of in-lens BSE detector with high-pass energy filter for the detection of low-loss backscattered electrons [21]. The Elstar column comes equipped with the through-the-lens detector (TLD), which works in four standard modes (detection of secondary electrons, detection of backscattered electrons, charge neutralization, and deep hole visibility). Selecting the display mode is performed by changing the potential of the auxiliary electrodes in the column. We will show that the TLD is designed to function as a band-pass filter for secondary electrons; this is achieved by using suitable potentials on the auxiliary electrodes. The detector collects secondary electrons over the narrow energy window which moves over the energy spectrum of secondary electrons. 

This SE band-pass filter is quick and easy to set up using software only, without the need of any hardware modification. Depending on its setting, it highlights either material composition differences or topography information in SE signal, or it can suppress charging artifacts in the image.

We have performed a large number of secondary electron trajectory simulations for the precise design of the Magellan microscope detection system. In the simulations, the electrostatic field of the detection system and the influence of the magnetic field of the objective lens were considered. The schema of the simulated layout, the simulation procedure, and the results will be described in the next section.

## 2. Materials and Methods 

### 2.1. Simulation Model

At present, a variety of commercial software for the simulations of the electron-optical properties of the system and ray tracing is available. Some programs are designed for computations of axially symmetric systems, others for 3D systems. In addition to selecting a suitable calculation program, it is crucial to include in the field calculations the exact geometry of the simulated system [1]. The simulations were done in commercially available software. The 3D electrostatic field of the system was calculated by Simion [22], which allows calculating the field of non-axial symmetrical arrangement. For magnetic field calculations and ray tracing in the combination of electrostatic and magnetic fields, we used program Electron Optical Design (EOD) [23]. 

**Figure 1:**
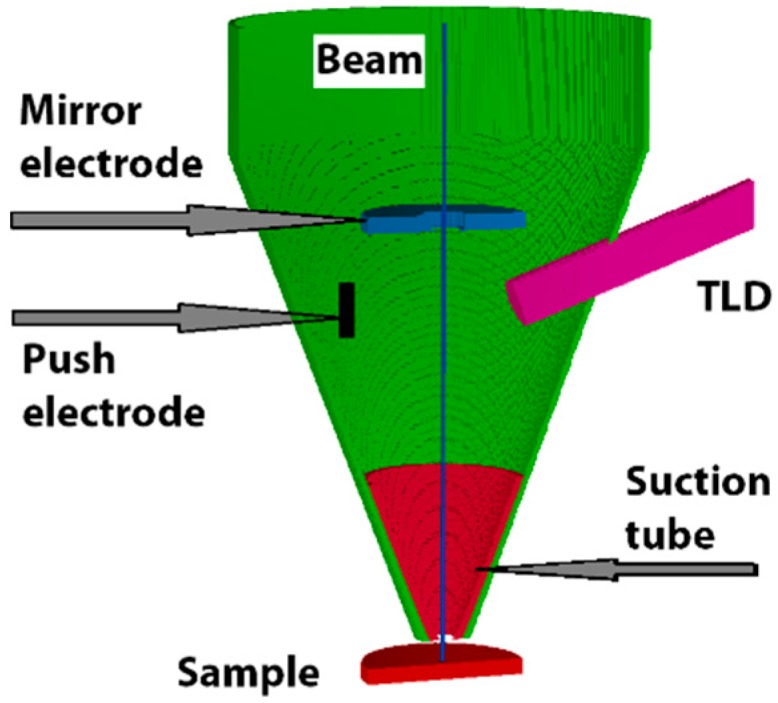
The arrangement used for simulation; through-the-lens detector and auxiliary electrodes inside the objective lens are shown schematically.

The cross-section of the 3D arrangement (Magellan 400 FEG SEM) [20] used in the simulation is shown in Figure 1. The through-the-lens detection system of secondary electrons consists of several electrodes to help SEs travel towards the scintillator of the TLD. The signal electrons move along the path between the sample and the detector in a magnetic and electrostatic field. The intensity of the magnetic field is determined by the primary beam energy, the working distance (WD), and the design of the objective lens. The intensity of the magnetic field cannot be changed arbitrarily, unlike the intensity of the electrostatic field, which can be varied by the bias on the auxiliary electrodes. At first, SEs emitted from the specimen are attracted into the objective lens (OL) by the positively biased “suction tube” electrode. The electrostatic field in the OL that is created by the “push electrode” and “mirror electrode” deflect SEs towards the scintillator. 

**Figure 2:**
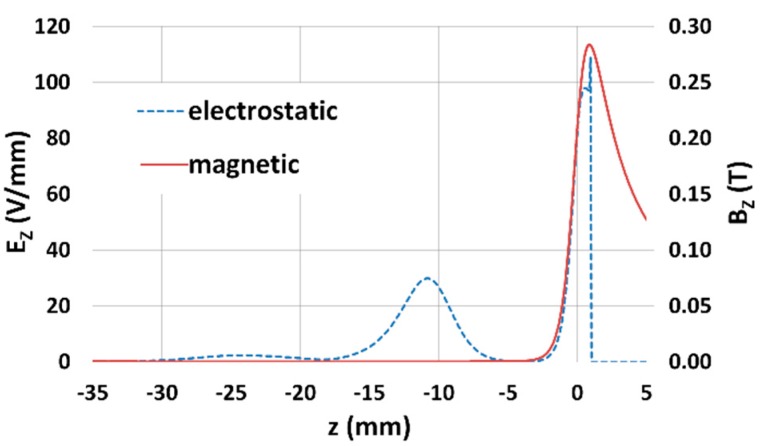
Axial electrostatic and magnetic field; WD = 1 mm, E_P_ = 1 keV, sample plane z = 1 mm.

Band-pass filter properties are simulated for fixed potentials of the suction tube (U_ST_ = +150 V), push electrode (U_PE_ = -140 V), mirror electrode (U_ME_ = +20 V) and scintillator (U_SC_ = +10 kV), the potential on the specimen varies from 0 V to 16 V, with a step of 4 V. The intensity of the magnetic field of the objective lens is determined by the primary beam energy and working distance. The magnetic field reaches the maximum in the proximity of the sample plane. The electrostatic field between the suction tube and sample attract secondary electrons to the objective lens, and simultaneously the magnetic field focuses electrons into the objective lens. In the objective lens, the intensity of the magnetic field is falling, and the trajectories of the secondary electron are influenced only by the electrostatic field. For a better idea of field distribution, Figure 2 shows the axial magnetic and electrostatic fields, and Table 1 illustrates the values of the field intensities in several *z* positions. 

### 2.2. Ray Tracing

Particle tracing is an essential part of determining the properties of a detection system. Knowing the detection properties allows us to set optimal imaging conditions while understanding the contrast in the final image. To obtain the relevant data, we need to simulate the corresponding number of trajectories. 

**Figure 3:**
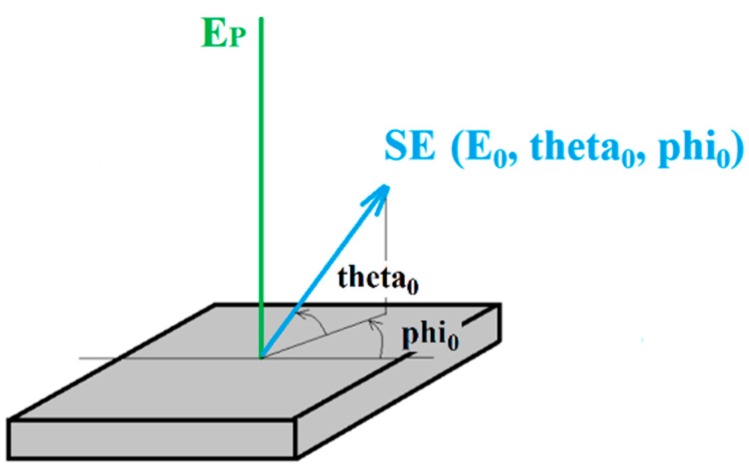
Angle definition of SE.

We traced secondary electrons on their way from sample to the TLD. It is necessary to trace a large bundle of particles to reach a high accuracy of particle tracing. SEs start in the center of the sample, the energy changing from 1 to 30 eV with a step of 1 eV, the polar angle from 0 to 90° with a step of 1° and the azimuthal angle from 0 to 360° with a step of 10° (see Figure 3). The TLD is asymmetrical to the optical axis; for that reason, we also study the azimuthal angle for SEs that escape the sample. 

**Figure 4:**
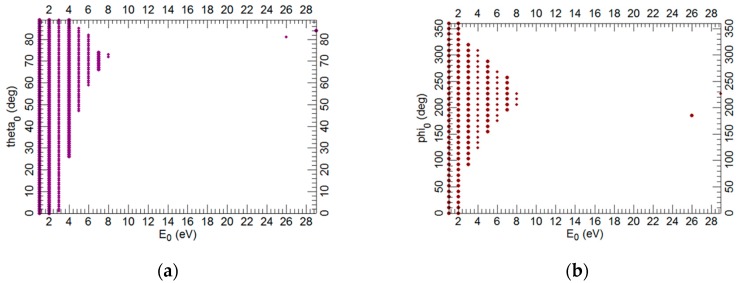
Acceptance diagrams. The initial polar angle theta_0_ (**a**) and initial azimuthal angle phi_0_ (**b**) as a function of the initial energy of electron E_0_, E_P_ = 1 keV, WD = 1 mm, U_SB_ = +4 V.

One of the possible outputs of ray tracing is the acceptance diagrams that show the detected secondary electrons depending on the selected parameters. In these simulations, we consider the detected secondary electrons of the first type (SE1: electron excited by the primary beam) and their initial polar as well as azimuthal angles (see Figure 4). 

**Figure 5:**
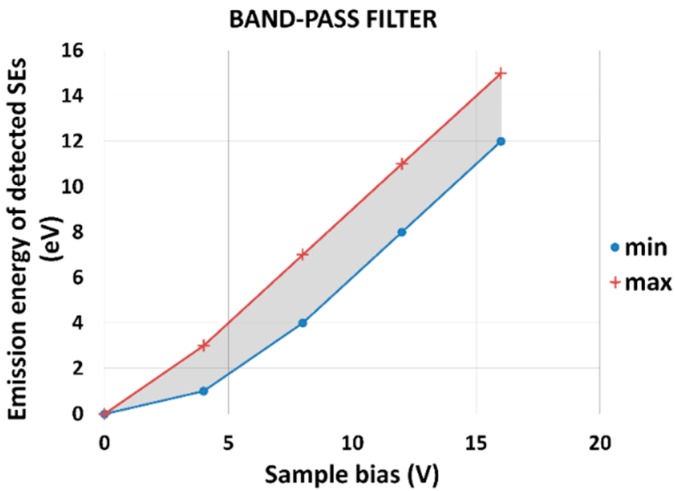
The final general graph for the band-pass filter. Emission energy of detected SEs as a function of the sample bias; E_P_ = 1 keV, WD = 1 mm.

For the description of a band-pass filter, we have to know initial polar angle theta_0_, first azimuthal angle phi_0_ and starting energy E_0_ of detected SEs1. We consider the cosine distribution of SE emission. The summarizing results of the band-pass filter simulations are plotted into one graph that best describes how the filter works. It is the dependence of the emission energy of SEs detected by the TLD, which takes into account the cosine distribution of emission on the positive sample bias (Figure 5). 

For each point on the curves in the graph, we have to trace nearly 100,000 particles. The blue curve (min) and the red curve (max) show the lower and upper threshold of the band-pass filter. The borders min and max mean that TLD detects SEs in the broad range of emitted polar and azimuthal angles. The results of the simulation of the band-pass filter shown in the graph in Figure 5 are presented numerically in Table 2. 

## 3. Results

### 3.1. Band-pass Filter–calculated Properties

**Figure 6:**
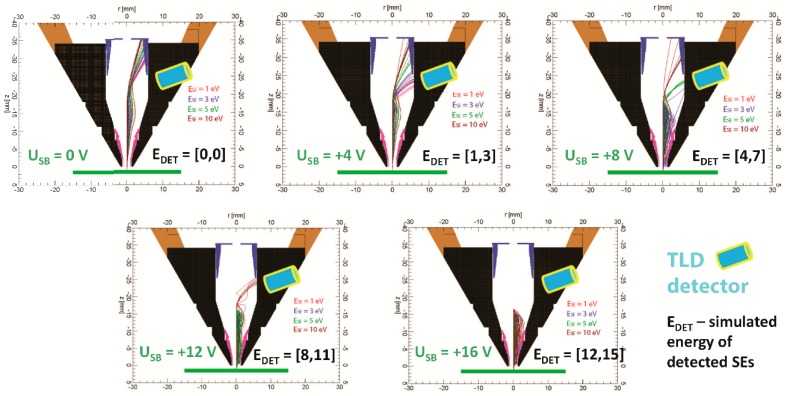
SE trajectories for energies E_SE_ = 1, 3, 5, 10 eV, polar angle theta_0_ = 0–90° with a step of 10°, azimuthal angle phi_0_ = 0°, sample bias U_SB_ = 0, +4, +8, +12, +16 V, E_P_ = 1 keV, WD = 1 mm.

**Figure 7:**
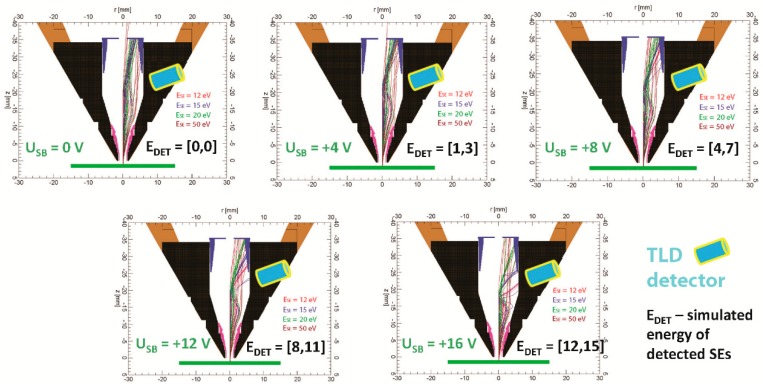
SE trajectories for energies E_SE_ = 12, 15, 20, 50 eV, polar angle theta_0_ = 0–90° with a step of 10°, azimuthal angle phi_0_ = 0°, sample bias U_SB_ = 0, +4, +8, +12, +16 V, E_P_ = 1 keV, WD = 1 mm.

The band-pass filter has a constant energy window of about 3 eV for sample bias from 4 to 16 V, but we have no signal of SE1 on the detector in simulations for sample bias 0 V. We simulated a small part of SEs1 to explain it, which should be visible in the central section of the simulation arrangement (Figure 6 and Figure 7). For sample bias 0 V, all SEs1 impact the mirror electrode or other parts inside the column are not detected by TLD. 

On the other hand, SEs3 emitted from the parts near the TLD should be detected. In the experiment for the sample bias U_SB_ = 0 V we can have an image of the specimen that is created mainly by the SE3 and a small portion of BSE.

Figure 6 and Figure 7 show that an increase of the sample bias U_SB_ will move trajectories of SE1 down to the suction tube electrode. It causes the detection of SE1 from some minimum energy E_DETMIN_ of SEs to maximum energy E_DETMAX_ of SEs, and it is the reason why TLD works as a band-pass filter. The differences between E_DETMAX_ and E_DETMIN_ gives the energy window of the band-pass filter. 

### 3.2. Verification of Simulations

In the previous paragraph, we present TLD, which works as a band-pass filter with the energy window of 3 eV. The next step is to confirm it by experiment on a specific selected specimen. For verification of simulation results, we chose a test sample with chromium and silver stripes on a silicon substrate. The width of the stripe was 3 µm in case of silver, and 6 µm for chromium for test sample A. For test sample B we used 1 µm Ag/2µm Cr. The thickness of both materials was 150 nm. The designed pattern was manufactured by a combination of e-beam lithography, wet etching, and lift-off technique.

**Figure 8:**
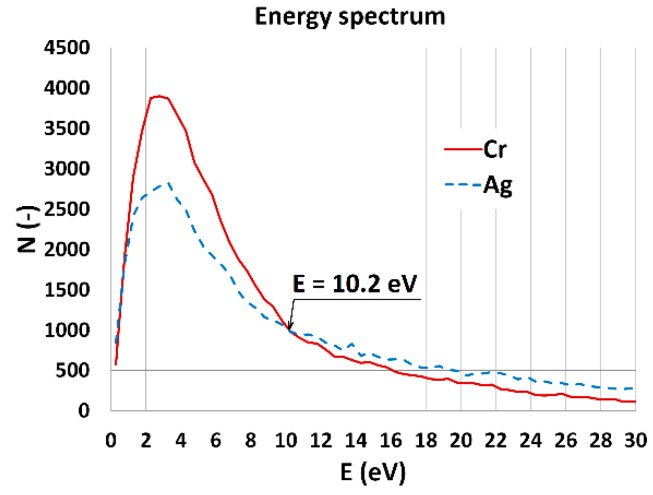
The simulated energy spectrum of secondary electrons for a chromium and silver specimen, primary beam energy E_P_ = 1 keV.

The energy spectrum of electrons emitted for both materials was simulated by using SW for Monte Carlo (MC) simulation of electron-specimen interaction in low-voltage SEM [24] based on the Geant 4 platform [25]. The primary beam energy was E_P_ = 1 keV and number of beam particles N(E_P_) = 10,000, were used in simulations. Results of MC simulations for SEs leaving the specimen are shown in Figure 8. 

The number of SEs emitted from chromium is higher than from silver for energies from 0 to 10.2 eV. However, for energies higher than 10.2 eV, more SEs are emitted from the silver specimen. If we know the simulated energy spectrum of SE for chromium and silver, we can set a band-pass filter to detect electrons from the chosen part of the energy spectrum. In this way we chose the detection electrons with energy lower or higher than the energy of the point of intersection of the curves, which in our case is E = 10.2 eV (see Figure 8), to obtain the contrast inversion.

The data in Table 3 shows how the band-pass filter works for our testing Ag/Cr sample. If we set sample bias in the range from 0 to 10 V, the filter detects electrons with energy from 1 to 9 eV. It means that we have more signal from the chromium stripe, which has to be brighter in an image. Once we set the sample bias from 14 to 16 V, SEs with energy detected from 10 to 15 eV, the Ag stripe becomes brighter. For sample bias from 11 to 13 V, secondary electrons with energy near the energy of the point of interaction (10.2 eV) are detected. It is a region of contrast inversion. 

One way to predict image contrast of a micrograph taken by the band-pass filter is to simulate an energy spectrum of secondary electrons, find the point of intersection, and then set the filter to detect electrons with lower as well as higher energies to study changes in image contrast. The graph in Figure 5 is general and independent of the sample material.

The second way to simulate the image contrast with applying the band-pass filter could be as follows: we can use the simulated energy spectrum for the studied material to ray trace through the band-pass filter system. We can then compare the number of detected electrons from each material for image contrast analysis. The results of the simulations for the Ag/Cr specimen are in Table 4. The number of the primary beam particle used for MC simulation is N_PE_ = 50,000, primary beam energy E_P_ = 1 keV. Number of emitted SEs and BSEs for Ag: N_SE_ + N_BSE_ = 86,756, for Cr: N_SE_ + N_BSE_ = 86,027. SEs and BSEs emitted from the sample are then traced through the band-pass filter arrangement. The number of detected particles and the collection efficiency (CE) of the detector is shown in Table 4. The changes in the image contrast are identical to the results in Table 3. 

### 3.3. Effect of Potential on the Suction Rube and the Mirror Electrodes

We focused on simulating the impact of the potential on the mirror and suction tube electrodes for a primary beam energy E_P_ = 1 keV and working distance WD = 1 mm. We changed the potential on the mirror electrode from −20 to +40 V with a step of 10 V. We adjusted the potential on the suction tube from the 0 to 270 V, namely the values of 0, 50, 100, 150, 200, and 270 V. The simulation results set limit values of the potential on these electrodes to maintain the filtering function of the secondary electrons. The potential on electrodes must be in the range of 10 to 40 V for mirror and 100 to 245 V for the suction tube. The increase of U_ME_ means reducing the width of the energy window of the filter. The potential on the suction tube U_ST_ slightly influences the width of the energy window, and the influence of a change in the potential is more visible at higher potentials on the sample. For the attainment optimal limit of the filter, concerning the small width of the energy window is appropriate for each sample bias set optimal potential on mirror and suction tube electrodes. 

### 3.4. Experiment

A series of microscope measurements were performed to verify the simulated filter properties. The experiment was performed in the Magellan 400 SEM with the test sample with chromium and silver stripes on a silicon substrate.

**Figure 9:**
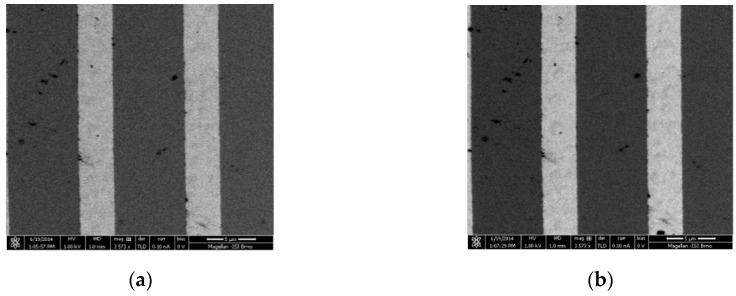
Ag (3 µm)/Cr (6 µm) stripes on a silicon substrate, without potential on the sample; standard TLD SE mode (**a**), BSE filtered image, without SEs1 (U_ST_ = −150 V) (**b**).

First, the test sample A was imaged by the TLD in standard SE mode (see Figure 9a). The Ag stripe is brighter than Cr, although the SE yield from both materials is almost the same. The contribution of SE3 causes it, which is mainly emitted from the mirror electrode after BSEs impact it. The atomic number of Ag, as well as Cr, is 47 and 24, respectively. BSE yield from the Ag stripe is higher than Cr. To confirm this assumption, we change the potential on the suction tube from +150 (standard TLD SE mode) to −150V. Thereby, only BSEs can go into OL, and the final contrast of micrograph is created by BSE signal (Figure 9b).

**Figure 10:**
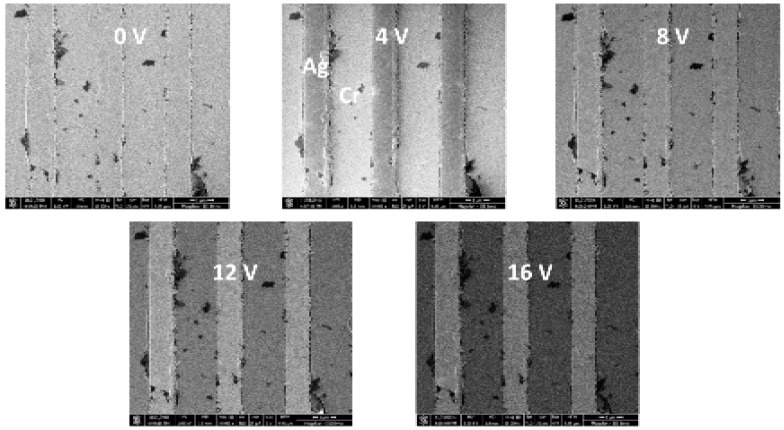
Ag (1µm)/Cr (2µm) stripes on a silicon substrate, the potential on the sample are 0, 4, 8, 12, and 16 V.

Second, we imaged the test sample B in the band-pass filter mode, for the sample bias, as used in the simulations. The micrograph of the sample was taken for positive potential on the specimen 0, 4, 8, 12, and 16 V. For the potential 4 V on the sample, the TLD detects mainly electrons with energies 1 to 3 eV, meaning that the SE yield of Cr is higher, and the stripes are brighter than Ag (see Figure 10). For the potential of 16 V, TLD detects SEs with energy from 12 to 15 eV. Consequently, the Ag strips are brighter. We can observe a change in contrast of the image depending on the sample potential. 

## 4. Discussion

As mentioned above, the TLD detector in the Magellan microscope can work as a band-pass filter without any modification of the system. All you have to do is set the appropriate potentials on the auxiliary electrodes. We use the microscope option to work in beam deceleration (BD) mode, which allows to change the bias on the sample. In band-pass filter mode, unlike BD mode, we attach a positive sample potential. For that reason, the method is suitable only for conductive samples.

The simulation model of the band-pass filter contains real geometry of the microscope, which is crucial for accurate calculation of the magnetic and electrostatic field distribution. The filter detection properties are determined from the result of the ray tracing of SEs1 emitted from the center of the sample. Simulation of the SE3 signal contribution to the detected signal has not been performed because the available software does not manage the process of BSE/SE3 conversion on solid surfaces. Anyway, the possible SE3 emission from the mirror electrode is suppressed due to the positive potential at that electrode and therefore the experimental data are not affected by the contribution of SE3 from the mirror electrode.

The MC simulations were performed by using the program of Kieft and Bosch [24] to work with lower electron energies. At low electron energy there are different approaches to the elastic and inelastic scattering method for each MC simulations in available programs that can give different results for examined materials [26].

The TLD detector detects a signal made up mainly of SEs1, which originate in close proximity to the sample surface and therefore it is necessary that the sample be clean, free of oxides and contaminating layer. For quantitative analysis, we use high purity samples without impurities that could cause a difference between experiment and simulated data for pure elements. The samples to be examined must be cleaned using standard mechanical and chemical methods prior to insertion into the microscope chamber [27]. Another source of contamination is from the microscope vacuum system. Plasma cleaning is very effective way of removing organic contamination. A new and effective technique to removing polymers and hydrocarbons is to use low-energy electron irradiation in a scanning low-energy electron microscope [28,29].

The band-pass filter is designed for primary beam energy 1 keV. In the range of units of keV various materials has their critical energy of electron impact at which the total emission equals to one, we have a non-charging mode [3].

Many optical devices such as an optical finite impulse response (FIR) filters, infinite impulse response (IIR) resonators, power splitters are based on a coated silicon slab [30]. The FIR filters are fabricated using an electron beam lithography and are experimentally examined by the scanning electron microscopes [31,32]. The band-pass filter for secondary electrons is powerful instrumentation to study the sample in its individual fabrication steps. 

## 5. Conclusions

The detection systems in SEMs, especially in-lens, offer a wide range of electron detection capabilities. These are complex systems with many electrodes whose electrostatic field in combination with the magnetic field influences electron trajectories differently. By suitable set-up of conditions, we get various detection modes, including energy filtering of secondary electrons. The properties of these systems can hardly be predicted without knowing the results of electron-optical simulations.

The ray tracing of secondary electrons in the model of the microscope Magellan 400 FEG SEM predicted that for appropriate potentials on the auxiliary electrodes inside the objective lens, the TLD detector works as a band-pass energy filter of SEs with the energy window of about 3 eV.

The simulated band-pass filter properties were verified experimentally on the test samples (Ag/Cr). The TLD in the band-pass filter mode predominantly detects secondary electrons of the first type, i.e., this method is susceptible to the purity or contamination of the sample surface. Energy filtration brings a change in contrast in the image as well as displaying details that are not otherwise visible. A significant advantage of the filter is the possibility to study the formation of the contrast in the image for the contamination of different metals and semiconductors. 

We presented a graph showing how the energies of the secondary electrons are detected by the band-pass filter, depending on the sample potential. This graph generally describes filter properties for a 1 mm working distance and primary energy of 1 keV, independent of the sample used. If we study the properties of a particular sample, we can simulate the energy spectrum of emitted electrons and trace these specific electrons that have energies and emission angles corresponding to the particular material. In this case, we also receive information about the detection efficiency of the detector for individual settings, plus we can quantitatively compare measured and simulated data. 

## Figures and Tables

**Table 1:** Axial intensity of the magnetic (B) and the electrostatic fields (E) in ***z*** position; WD = 1 mm, E_P_ = 1 keV.

z position (mm)	1	0	−10	−20
Axial mg. field B (mT)	290	214	309 × 10^-3^	203 × 10^-3^
Axial el. field E (V/mm)	0	81.9	26.9	1.3

**Table 2:** The emission energy of SEs detected by the TLD for sample bias 0, 4, 8, 12 and 16 V, working distance WD = 1 mm, primary beam energy E_P_ = 1 keV (data from the graph in Figure 5).

Sample Bias (V)	Energy Window Detected by TLD (eV)	
	min	max
0	0	0
1	0	1
2	0	1
3	1	2
4	1	3
5	2	4
6	3	5
7	4	6
8	4	7
9	5	8
10	6	9
11	7	10
12	8	11
13	9	12
14	10	13
15	11	14
16	12	15

**Table 3:** The simulated band-pass filter properties for Ag/Cr sample.

Sample Bias (V)	Energy of Detected Electrons (eV)	Image Contrast Information
0 – 10	1 – 9	Cr - brighter
11 – 13	7 – 12	CONTRAST INVERSION
14 – 16	10 – 15	Ag - brighter

**Table 4:** Simulated results for the specimen Ag/Cr; the number of traced particles of Ag, as well as Cr, is 86,756 and 86,027, respectively.

Sample Bias (V)	Number of Detected Particles by TLD (-)		Collection Efficiency of the TLD (%)	
	Ag	Cr	Ag	Cr
0	328	423	0.4	0.5
4	2024	2405	2.3	2.8
8	10,500	13,258	12.1	15.4
12	5674	6254	6.5	7.3
16	4413	4232	5.1	4.9
